# Identification of testicular cancer immune infiltrates and novel immune cell subtypes

**DOI:** 10.1002/2211-5463.13688

**Published:** 2023-08-10

**Authors:** Zhiguo Zhu, Xujun Xuan, Xinkun Wang, Miaomiao Wang, Chunyang Meng, Zhonghai Li

**Affiliations:** ^1^ Department of Urology, Affiliated Hospital of Jining Medical University Jining Medical University China; ^2^ Postdoctoral Mobile Station of Shandong University of Traditional Chinese Medicine Jining China; ^3^ Department of Andrology, The Seventh Affiliated Hospital Sun Yet‐sen University Shenzhen China; ^4^ Department of Medical, Affiliated Hospital of Jining Medical University Jining Medical University China; ^5^ Medical Research Center, Affiliated Hospital of Jining Medical University Jining Medical University China

**Keywords:** biomarker, immune infiltration, molecular subtyping, risk signature, STC1, testicular germ cell tumors

## Abstract

Testicular germ cell tumors (TGCT) are the most common type of testicular cancer, comprising 90–95% of cases and representing the most prevalent solid malignancy in young adult men. Immune infiltrates play important regulatory roles in tumors, but their role in TGCT remains unclear. Molecular subtyping is a promising way to provide precisely personalized treatment and avoid unnecessary toxicities. This study investigated immune infiltrates, key biomarkers, and immune subtyping of TGCT. In GSE3218, 24 differentially expressed immune genes (immDEGs) were identified. A new risk signature consisting of six immDEGs was developed using these genes. Individuals in the high‐risk group had poor overall survival (OS; hazard ratio of 4.61 and *P*‐value < 0.001). We validated the six‐immDEGs risk signature in pure seminoma and mixed TGCT types. Two distinct immune patterns (Cluster 1 and Cluster 2) were identified using the consensusclusterplus, and Cluster 1 possessed an unfavorable OS compared with Cluster 2 (hazard ratio, 2.56; *P* < 0.001). Cluster 1 patients had significantly lower naive B cells, memory B cells, plasma cells, naive CD4 T cells, gamma delta T cells, and activated dendritic cells than Cluster 2 patients. Genes relating to the WNT signaling pathway, TGF‐β signaling pathway, antigen processing and presentation, and NK cell‐mediated cytotoxicity were associated with TGCT. STC1 was elevated in TGCT tissues, and its high expression showed advanced clinicopathological characteristics and poor prognosis of TGCT. Our findings may contribute to an increased understanding of the onset and progression of TGCT.

## Graphical abstract

In this study, a risk signature comprising immune‐related genes was successfully developed and shown to be effective in predicting the prognosis of patients with testicular germ cell tumors (TGCT). We also identified testicular cancer immune infiltrates and novel immune cell subtypes. These findings may aid in comprehending the onset and progression of TGCT and devising efficient treatment approaches.

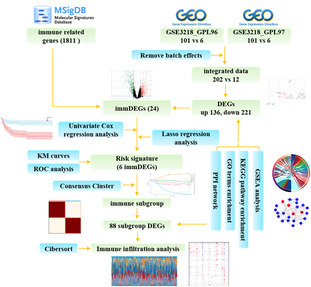

**Abbreviations**
BPs: biological processes
CCs: cellular components
DEGs: differentially expressed genes
FC: fold change
GEO: gene expression omnibus
GO: gene ontology
GSEA: gene set enrichment analysis
H&E: hematoxylin and eosin
IHC: immunohistochemical
immDEGs: differentially expressed immune genes
IRS: immunoreactivity score
KEGG: Kyoto Encyclopedia of Genes and Genome
KM: Kaplan–Meier
LASSO: least absolute shrinkage and selection operator
MFs: molecular functions
OS: overall survival
PD‐L1: programmed death receptor ligand‐1
PPI: protein–protein interaction
ROC: receiver operating characteristic
STC1: stanniocalcin‐1
STRING: Search Tool for the Retrieval of Interacting Genes
TGCT: testicular germ cell tumors
TME: tumor microenvironment
UMAP: uniform manifold approximation and projection

Testicular cancer is the most prevalent solid malignancy among young adult men [1, 2]. The incidence has been steadily increasing, especially in the developed countries [3]. The etiology of testicular cancer is still unclear, and its pathological types are diverse, most of which (90–95%) are testicular germ cell tumors (TGCT). Most of these patients are cured with surgery alone or in combination with chemotherapy, with a 5‐year survival rate of 98% for localized disease. However, primary resistance, disease progression after therapy, and adverse effects of treatment are still major clinical challenges [4, 5]. Therefore, identifying the molecular mechanism and novel predictors of prognosis is important for diagnosis and personalized therapy.

The immune cells in the tumor microenvironment (TME) are critically involved in tumorigenesis [6, 7], and effective immunotherapy has been achieved in multiple tumors [6]. Due to the existence of the blood‐testis barrier, the testis is often regarded as a special site that is exempt from normal systemic immune surveillance. However, immune infiltrates are widespread in testicular tumors, particularly in TGCT. In 2002, Yakirevich *et al*. [8] found that the number of activated cytotoxic lymphocytes was increased in testicular seminomas. In 2015, Fankhauser *et al*. [9] highlighted that programmed death receptor ligand‐1 (PD‐L1) was frequently expressed in TGCT. Subsequent studies also confirmed PD‐1 (programmed death 1)/PD‐L1 as a potential therapeutic target of TGCT [10, 11, 12, 13]. Although the immune infiltrate plays an important role in TGCT, it remains scarcely studied compared with other tumors. Especially, a systematic understanding of the immune milieu in TGCT is currently lacking.

The present research employed gene expression profiles to assess immune‐related genes that exhibited a significant difference between individuals with TGCT and control samples. A risk signature comprising immune‐related genes was successfully developed and is effective in predicting patient prognosis. Moreover, two distinct immune patterns with significant prognostic differences were identified. The identification of variations in immune infiltration patterns between TGCT and healthy tissues would aid in comprehending the onset and progression of TGCT and devising efficient treatment approaches.

## Materials and methods

### Data and human tissues collection

The datasets GSE3218 (GPL96) and GSE3218 (GPL97) were provided by the Gene Expression Omnibus (GEO) database [14]. To generate the integrated GEO dataset, Empirical Bayes methods were employed to adjust batch effects in microarray expression data [15]. The merged data contained 202 TCGT samples and 12 normal samples. TCGA dataset was downloaded from the assistant for clinical bioinformatics database platform (https://www.aclbi.com/). The MSigDB databases were used to obtain 1811 immune‐related genes [16].

A total of 24 TGCT (seminoma) tissues were retrieved from January 2016 to September 2022 in Affiliated Hospital of Jining Medical University. And normal testicular tissues (*n* = 8) were obtained from patients with prostate cancer undergoing orchiectomy from January 2018 to January 2019. Two pathologists confirmed all tissue types using hematoxylin and eosin (H&E) staining. The study was conducted in accordance with the Declaration of Helsinki. All individuals granted their written informed consent prior to participation. The approval of this study was granted by the Ethics Committee of the Affiliated Hospital of Jining Medical University (number: 2023‐04‐C038).

### Identification of differentially expressed immune genes

Differentially expressed genes (DEGs) between tumors and control samples were identified utilizing the ‘limma’ r package [17] (cutoff criteria, false discovery rate < 0.05 and |fold change| > 2). Volcano plots and heat maps were utilized to visualize the outcomes of DEGs. In order to identify the differentially expressed immune genes (immDEGs), the DEGs and immune‐related genes were intersected.

### Construction of the immune‐related genes risk signature

Univariate Cox regression analysis was conducted to identify immDEGs that were related to overall survival (OS). The r package ‘glmnet’ [18] and least absolute shrinkage and selection operator (LASSO) were applied to obtain the characteristic genes of risk signature. The calculation formula of the model is as follows: risk score = sum (each gene's expression × corresponding coefficient). To classify individuals into high‐ and low‐risk groups, the median risk score was used as a cutoff value. The Kaplan–Meier (KM) method was utilized to examine differences in OS between both risk groups. Time‐dependent receiver operating characteristic (ROC) curves were generated to assess the stability of the risk signature at 1‐, 3‐, and 5‐year durations.

### Identification of immune molecular subtypes and extraction of DEGs between two distinct immune patterns

The r package ‘consensusclusterplus’ [19] was employed to identify the immune molecular subtypes. The KM method was employed to analyze OS differences between immune molecular subtypes. The r package ‘limma’ was utilized to extract DEGs between two distinct immune patterns (cutoff criteria, false discovery rate < 0.05, and |fold change (FC)| > 1.5). The ROC curve was used to evaluate the ability of genes to distinguish immune subgroups.

### Construction of PPI network

The Search Tool for the Retrieval of Interacting Genes [20] (STRING) was utilized to construct the protein–protein interaction (PPI) network, which was then visualized using the cytoscape software (https://cytoscape.org/).

### Enrichment analysis

For gene ontology (GO) annotation analysis in terms of molecular functions (MFs), biological processes (BPs), and cellular components (CCs), as well as Kyoto Encyclopedia of Genes and Genome (KEGG) pathway enrichment analysis, the r packages ‘org.hs.eg.db’ and ‘clusterprofiler’ [21] were employed. For statistical significance, a *P*‐value of < 0.05 was set as the criterion. To explore the variations in BPs between distinct subgroups, gene set enrichment analysis (GSEA) [22] was carried out. To assess relevant pathways and underlying mechanisms, the ‘c2.cp.kegg.v7.4.symbols.gmt’ gene sets were downloaded from the Molecular Signatures Database. A *P*‐value of < 0.05 was taken to be a statistically significant criterion for this analysis.

### Comparison of immune infiltrate cells

The analytical tool CIBERSORTx [23] can use gene expression data to perform linear support vector regression, thus estimating immune cell infiltration. We calculated 22 immune cell types in patients using CIBERSORTx and analyzed the correlation and difference between the proportion of different immune cells, the proportion of immune cells, and gene expression. A statistically significant *P*‐value of < 0.05 was used to determine the significance of the results.

### Immunohistochemical assessment

The TGCT and normal testicular tissues were subjected to an immunohistochemical (IHC) assay using a 1 : 100 dilution of an antibody against STC1 (#20621‐1‐AP; Proteintech, Wuhan, Hubei, China). Paraffin wax was used for embedding the tissues. IHC was employed according to a standard method. A final immunoreactivity score (IRS) was obtained for each case by multiplying the positive cell percentage score (< 5%, 0; 5–25%, 1; 26–50%, 2; 51–75%, 3; > 75%, 4) and the intensity score (negative, 0; weak, 1; moderate, 2; strong, 3). Divided into four levels: 0–2: negative (−), 3–5: weak positive (+), 6–8: positive (++), 9–12: strong positive (+++).

### Statistical analysis

All the statistical analyses were carried out utilizing rstudio 4.1.3 (Posit, BOSTON, MA, USA). The independent Student's *t*‐test (normally distributed variables) and Mann–Whitney *U*‐test (non‐normally distributed variables) were conducted to compare two groups of continuous variables. Pearson correlation analysis was conducted to estimate correlation coefficients between distinct variables. A *P*‐value of < 0.05 suggested the significance level. Other statistical methods have been mentioned in the corresponding ‘Materials and methods’ section above.

## Results

### Dysregulation of immune‐related genes in TGCT

**Fig. 1:**
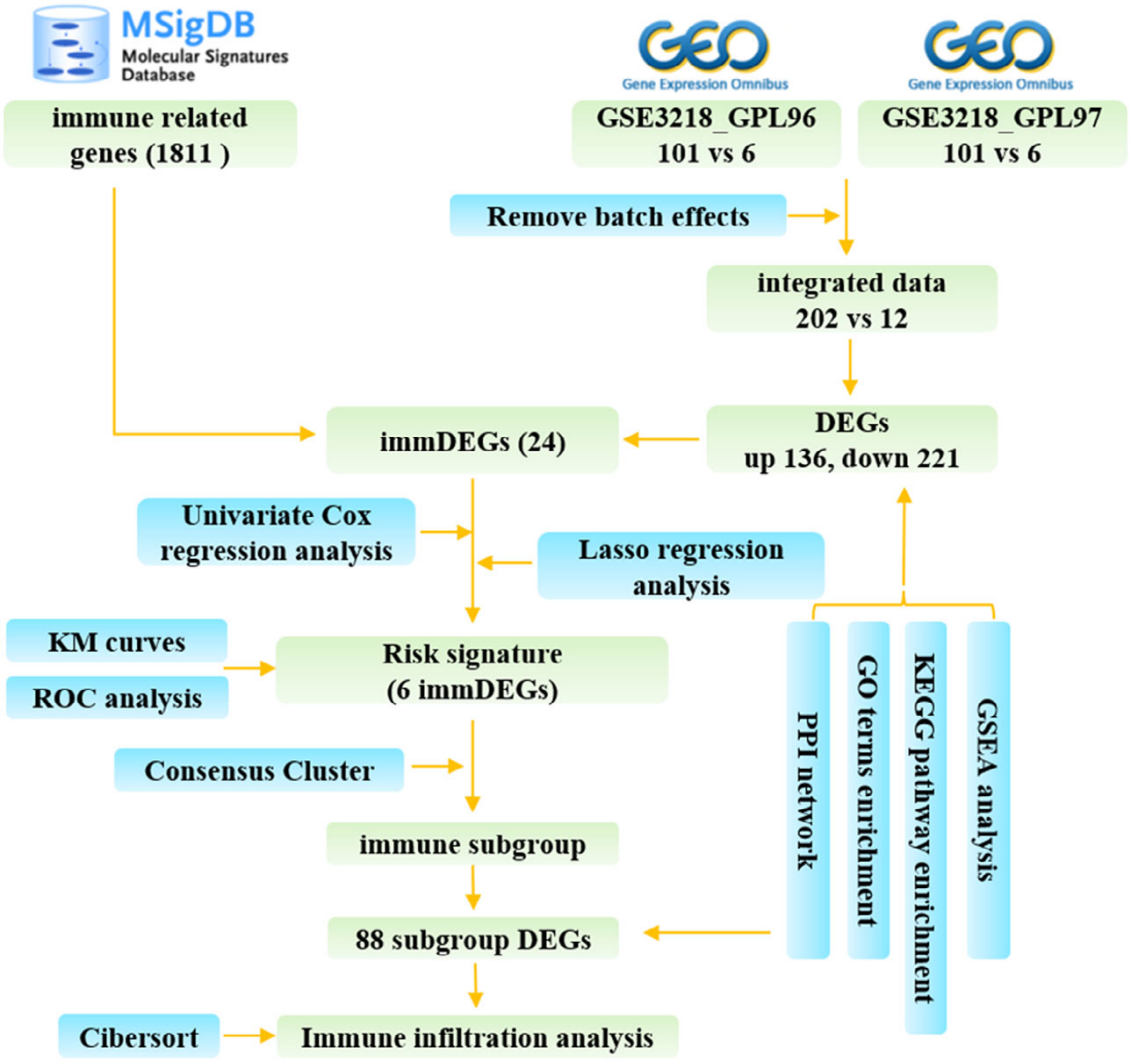
Study flow diagram.

**Fig. 2:**
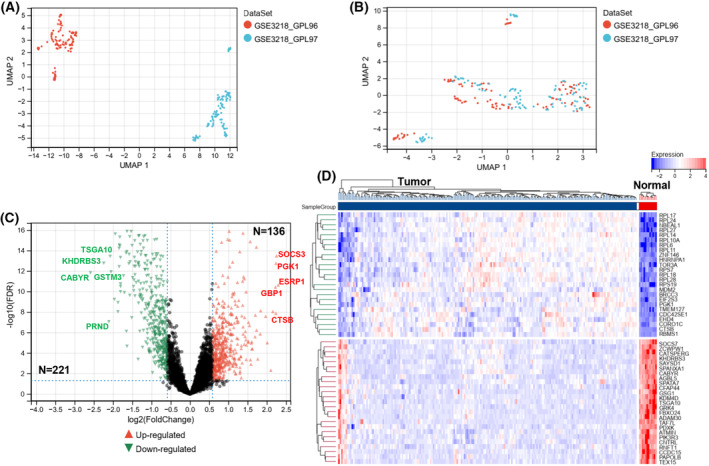
Identification of DEGs. Uniform manifold approximation and projection (UMAP) between datasets before de‐batching (A) and after de‐batching (B). (C) The volcano plot was constructed based on fold change values and *P*‐adjust, with red and blue dots representing upregulated and downregulated genes, respectively. (D) The heatmap depicting the top 50 genes identified through analysis of differential gene expression.

**Fig. 3:**
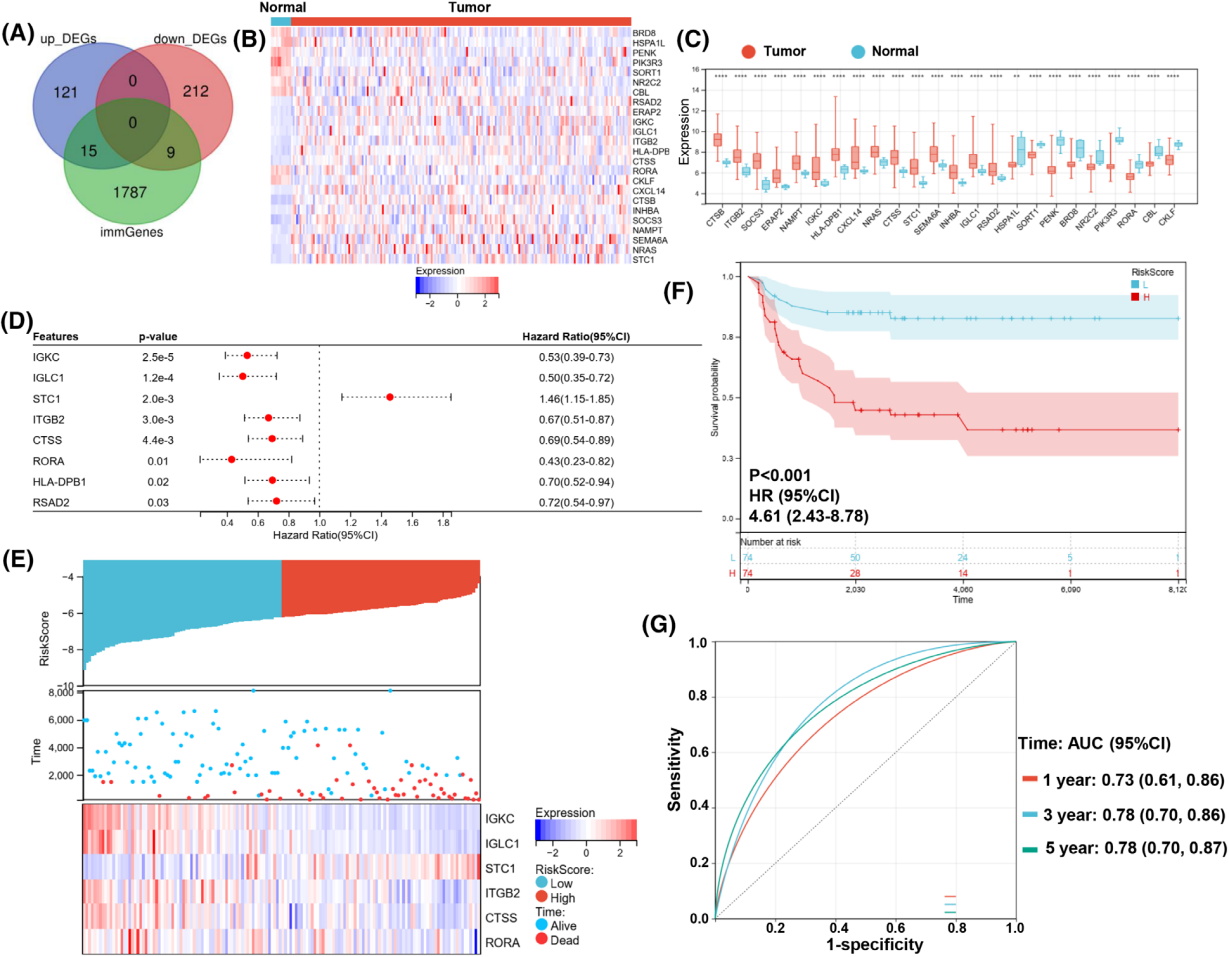
Differentially expressed immune genes and construction of the immune‐related genes risk signature. (A) Immune gene vs. DEG Venn diagram. (B) The heatmap of 24 immDEGs. (C) The expression histogram of 24 immDEGs in TGCT (*n* = 202) and normal tissues (*n* = 12). (D) Identification of eight immDEGs associated with OS of TGCT via univariate Cox regression. (E) The risk score, survival time, and survival status of TGCT. (F) Kaplan–Meier survival analysis of the two risk groups, TGCT samples were divided into high‐ and low‐risk groups based on their median risk score. (G) Time‐dependent ROC curves verified the predictive efficacy of the risk signature at 1, 3, and 5 years. Presentation of data as means ± standard deviation. **P < 0.01; ****P < 0.0001; rank‐sum test. immGenes, immune genes.

Figure 1 presents the flow chart for data collection and analysis. The integrated GEO dataset, including 202 TCGT samples and 12 normal samples, was obtained after removing batch effects (Fig. S1; Fig. 2A,B). The result of unsupervised clustering of the samples with all genes is shown in Fig. S2A. In total, 357 DEGs were screened out in TCGT samples compared with normal samples. Out of these, 136 were upregulated, whereas 221 were downregulated (Fig. 2C,D). The five most significantly upregulated genes were *ESRP1* (FC = 5.42), *SOCS3* (FC = 4.92), *PGK1* (FC = 4.77), *CTSB* (FC = 4.72), and *GBP1* (FC = 4.71). The five most significantly downregulated genes were *CABYR* (FC = 0.06), *KHDRBS3* (FC = 0.08), *PRND* (FC = 0.10), *TSGA10* (FC = 0.12), and *GSTM3* (FC = 0.13). By intersecting DEGs with immune‐related genes, a total of 24 immDEGs were identified. Among these, 15 were upregulated, while nine were downregulated (Fig. 3A). The heat maps (Fig. 3B) and histograms (Fig. 3C) show their expression levels in samples.

### Construction of the immune‐related genes risk signature

If the risk signature contains too many variables, it will increase the difficulty of applying the signature. Therefore, we first screened for genes that were associated with patients' survival time. A total of eight immDEGs that were linked to the OS of patients were identified via univariate Cox regression (Fig. 3D). Except for *STC1*, other genes served as protective factors. But only the expression of *RORA* was decreased in TGCT tissues compared with its corresponding normal tissues. To prevent overfitting of the model, the LASSO algorithm was utilized to discover six characteristic genes out of eight immDEGs (Fig. S3).

$$\text{Risk Score}=\begin{array}{l} \left(-0.36\right)\times \text{IGKC}+\left(-0.20\right)\times \text{IGLC1}+0.45\\ \times \text{STC1}+\left(-0.05\right)\times \text{ITGB2}+\left(-0.02\right)\\ \times \text{CTSS}+\left(-0.93\right)\times \text{RORA.} \end{array}$$

As per the median risk score calculated using the formula, TGCT samples were categorized into high‐ and low‐risk groups (Fig. 3E). The scatter plot depicted the survival rates of individuals based on their risk scores, and the heatmap demonstrated the differential expression profiles of six immDEGs between both risk groups. KM survival curves revealed that the individuals in the high‐risk group possessed a poor OS (HR, 4.61; 95% CI, 2.43–8.78; *P* < 0.001; Fig. 3F). The efficacy of the risk signature was validated using time‐dependent ROC curves at 1‐, 3‐, and 5‐year durations. (Fig. 3G).

**Fig. 4:**
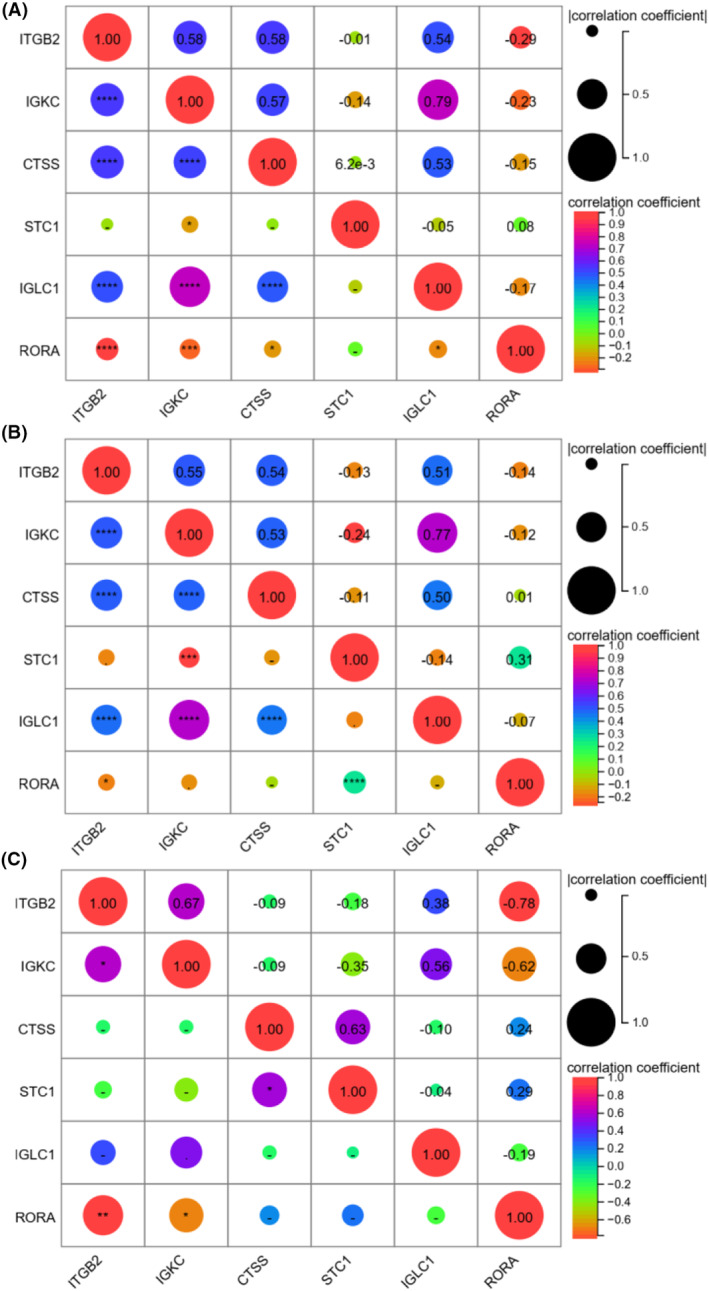
Correlations between gene expression levels between six immDEGs in all samples (A), TGCT sample (B), and normal samples (C).

Correlations between gene expression levels between six immDEGs were examined in all samples (Fig. 4A), TGCT samples (Fig. 4B), and normal samples (Fig. 4C). *STC1*, the only hazardous gene, had weak correlations with other genes in all samples. However, *STC1* had positive correlations with *RORA* and *CTSS* and a negative correlation with *IGKC* in subgroups. There was a clear correlation between *IGKC*, *IGLC1*, *ITGB2*, *CTSS*, and *RORA* in different groups. The collections between *CTSS* and *IGKC*, *CTSS* and *ITGB2*, *RORA*, and *ITGB2*, and *RORA* and *IGKC* showed considerable variation in TGCA and normal samples.

### Identification of immune molecular subtypes

**Fig. 5:**
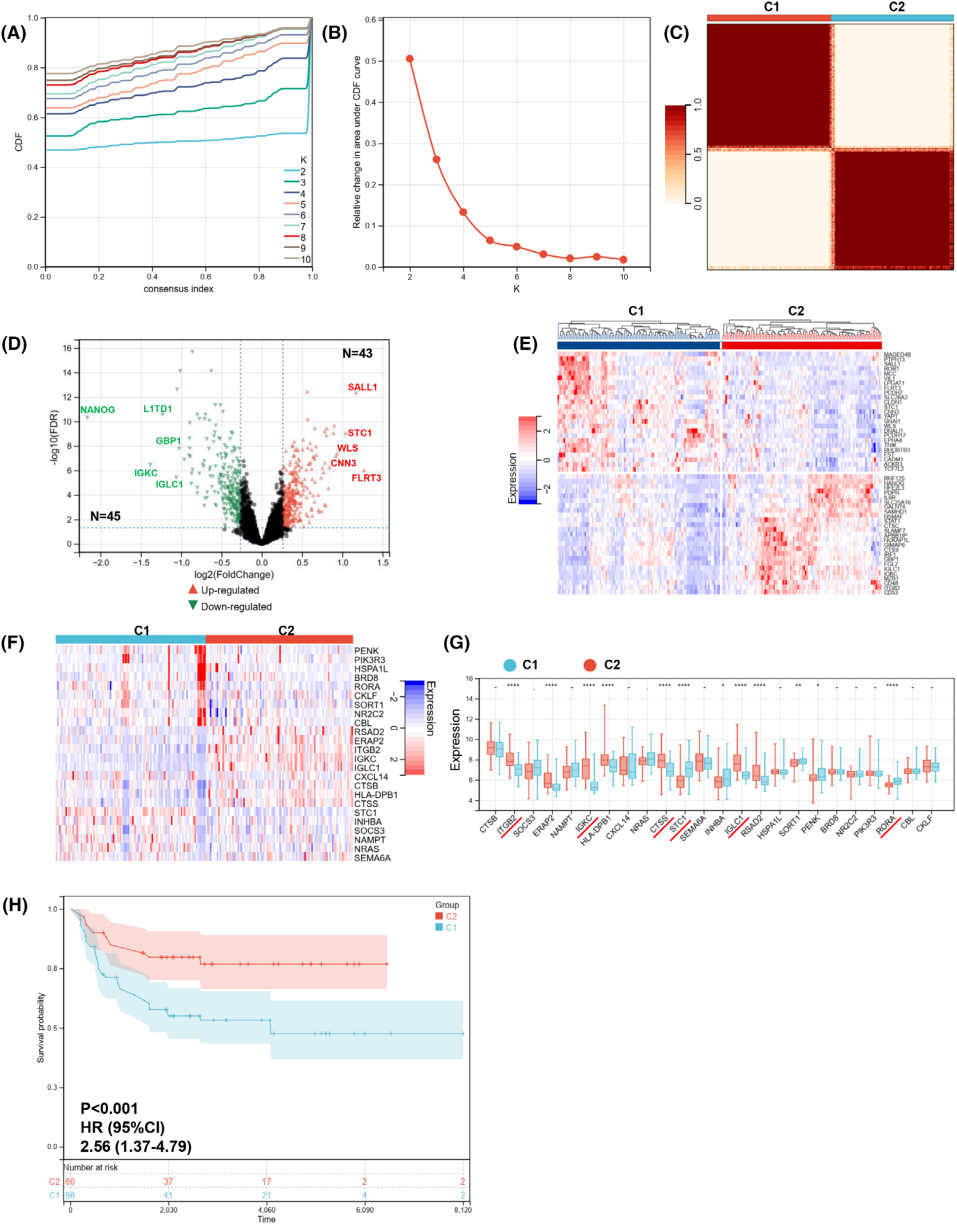
Identification of immune molecular subtypes. (A–C) Two immune molecular subtypes were obtained using consensusclusterplus. (D) DEGs between immune molecular subtypes visualized by volcano plot. (E) A heatmap depicting the top 50 genes identified through analysis of differential gene expression. (F) The heatmap of 24 immDEGs. (G) The expression histogram of 24 immDEGs in two immune molecular subtypes (C1, *n* = 108; C2, *n* = 106). (H) Kaplan–Meier survival analysis of the two immune molecular subtypes. Presentation of data as means ± standard deviation. *P < 0.05; **P < 0.01; ****P < 0.0001; rank‐sum test.

**Fig. 6:**
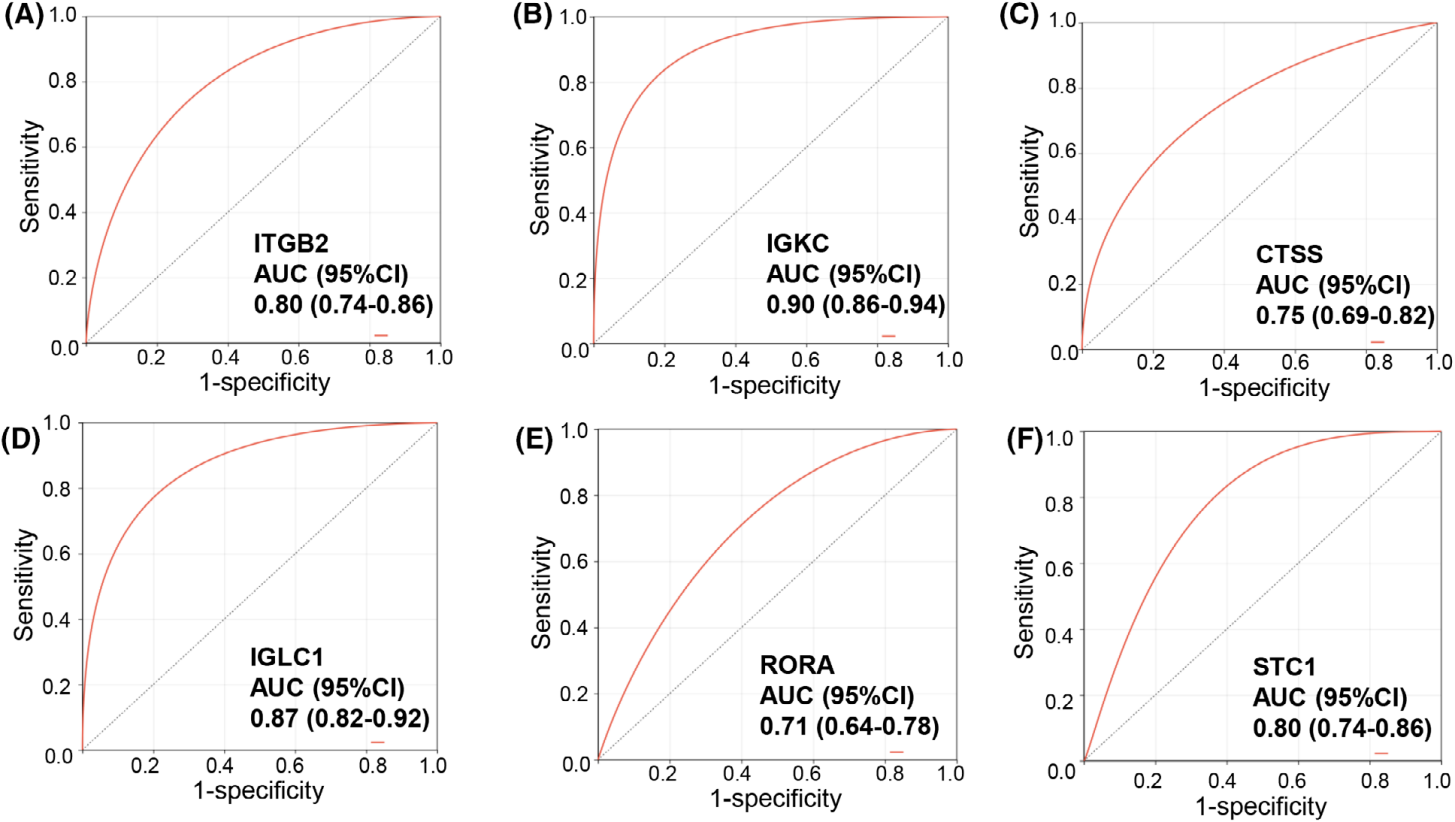
ROC curves of six characterized genes demonstrating their ability to distinguish between Cluster 1 and Cluster 2 independently. ROC curves of ITGB2 (A), IGKC (B), CTSS (C), IGLC1 (D), RORA (E), and STC1 (F).

Based on six signature immDEGs, two immune molecular subtypes (Cluster 1 and Cluster 2) were identified utilizing the r package ‘consensusclusterplus’ (Fig. 5A–C). Cluster 1 comprised 108 samples, and Cluster 2 comprised 106 samples. When comparing Cluster 1 samples with Cluster 2 samples, 88 DEGs were determined, with 43 upregulated and 45 downregulated DEGs (Fig. 5D). Figure 5E shows the top 25 DEGs to show the considerable differences between the two clusters. Figure 5F shows 24 immDEGs expression levels between the two groupings. Expression levels of *ITGB2*, *ERAP2*, *IGKC*, *HLA‐DPB1*, *CTSS*, *IGLC1*, and *RSAD2* were remarkably elevated in Cluster 2 than that in Cluster 1, whereas the expression levels of STC1, INHBA, SORT1, PENK, and RORA were considerably lowered in Cluster 2 as opposed to Cluster 1 (Fig. 5G). Moreover, KM survival curves revealed that Cluster 1 possessed a poor OS (HR, 2.56; 95% CI, 1.37–4.79; *P* < 0.001; Fig. 5H). The ROC curves were used to assess the six signature immDEGs individually predicted values. The findings showed that all six signature immDEGs had good classification efficacy for two immune molecular subtypes (Fig. 6).

### PPI network and enrichment analysis

**Fig. 7:**
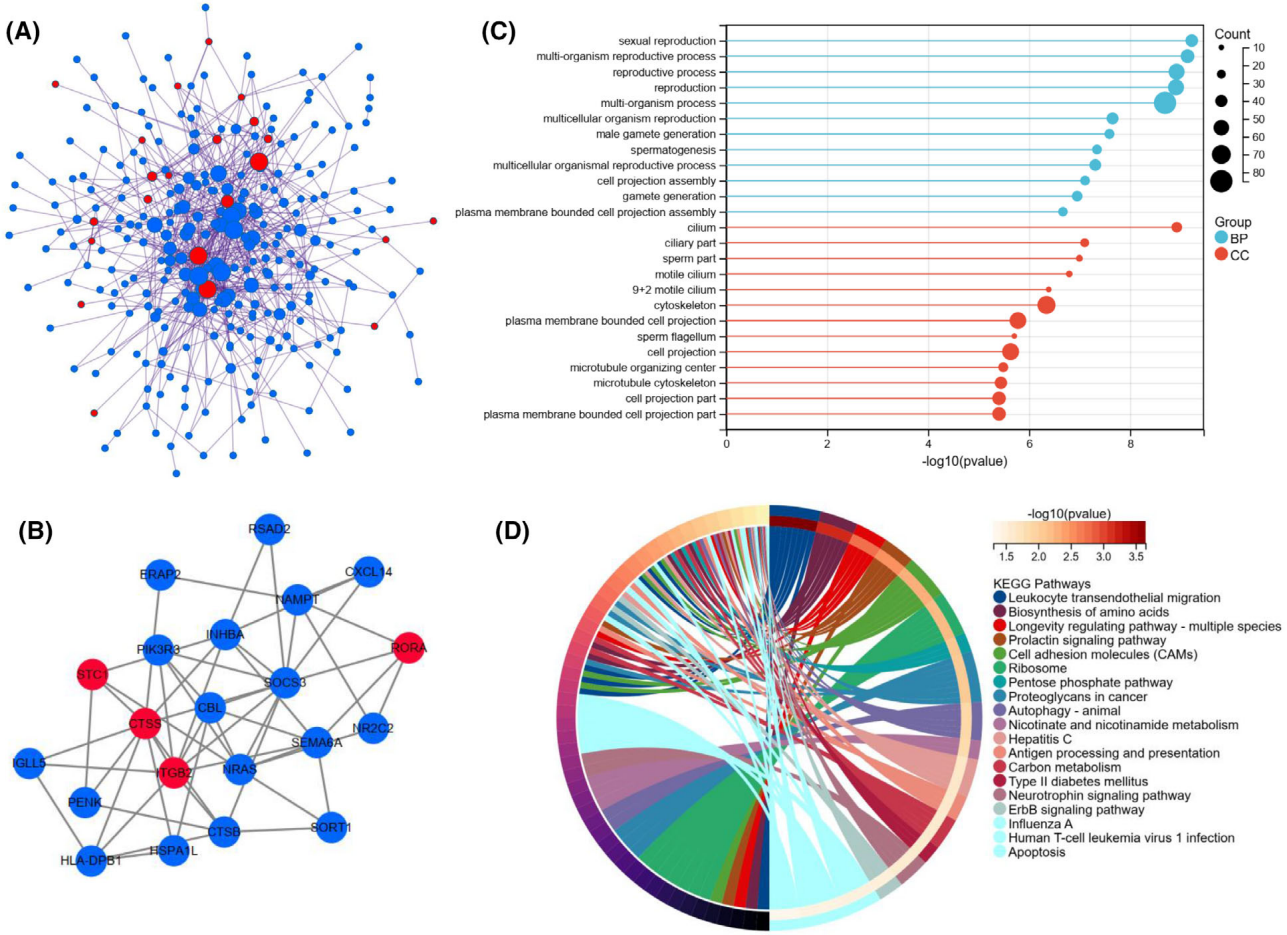
PPI network and functional analysis of DEGs between TGCT and normal samples. (A) DGEs PPI network: red nodes indicate immDEGs. (B) ImmDEGs PPI network: red nodes indicate characterized genes of risk signature. (C) GO functional enrichment analysis. (D) KEGG functional enrichment analysis.

A PPI network of DEGs (tumor vs. normal) was constructed using cytoscape software, according to the data from the STRING database, to explore their relationships. The PPI network of DEGs demonstrated that the distribution of the 24 immDEGs was relatively scattered (Fig. 7A). In the PPI network of immDEGs, *STC1* and *RORA* were linked to four immDEGs, ITGB2 was related to nine immDEGs, and CTSS was linked to 10 immDEGs (Fig. 7B). Following the initial analysis, the role of DEGs between TGCT and normal samples in biological functions was investigated. Initially, the DEGs were found to have a significant association with fertility (Fig. 7C). Subsequently, KEGG pathway analysis revealed enrichment of these DEGs in immune, aging, cancer, and infection‐related pathways (Fig. 7D).

**Fig. 8:**
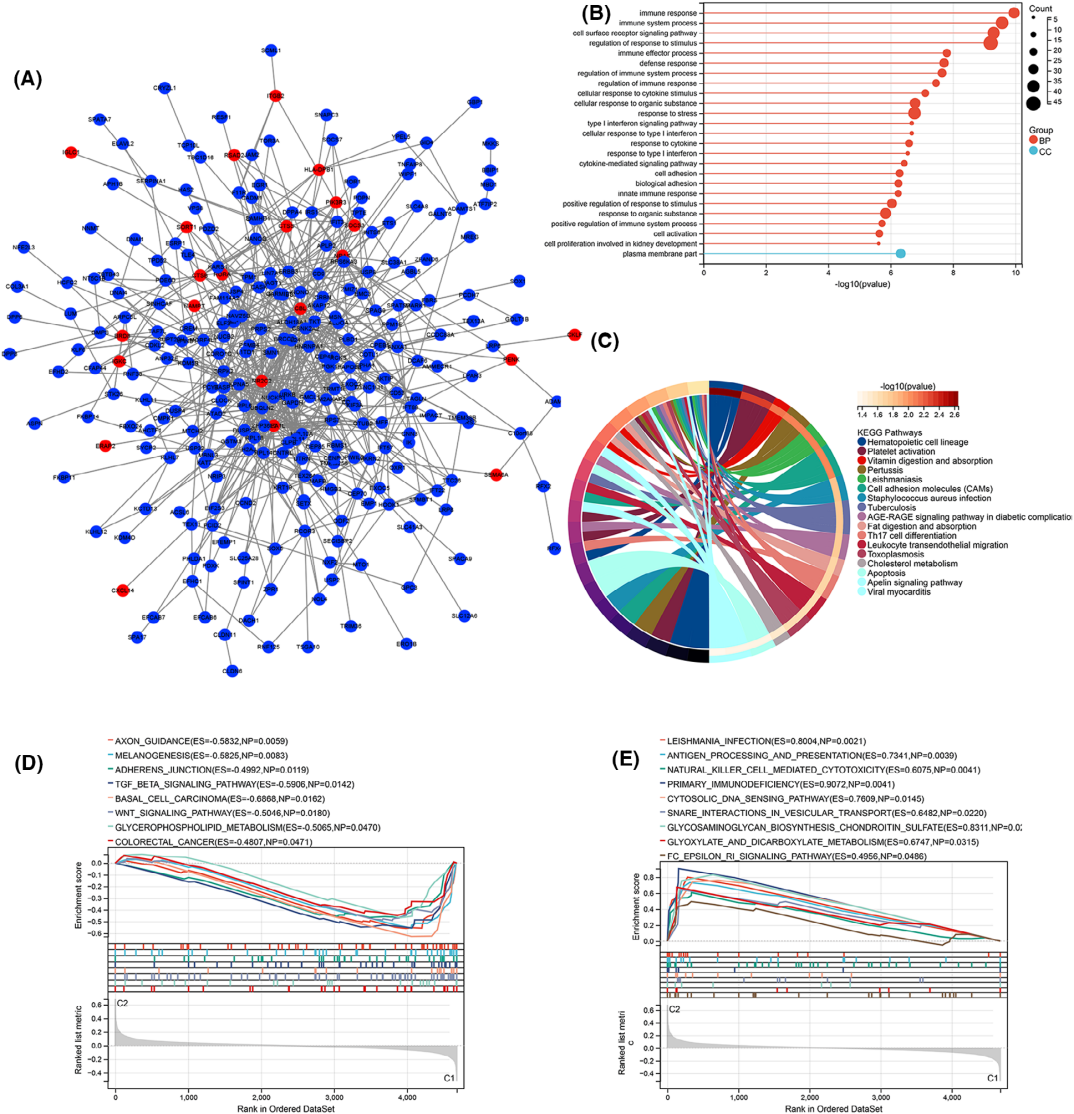
PPI network and functional analysis of DEGs and GSEA analysis between two different immune patterns. (A) DGEs PPI network: red nodes indicate immDEGs. (B) GO functional enrichment analysis. (C) KEGG functional enrichment analysis. Activated (D) and inhibited (E) BPs in Cluster 1 compared with Cluster 2.

A PPI network was constructed for the DEGs between the two immune patterns (Fig. 8A). Similarly, the distribution of 24 immDEGs was relatively scattered. The outcomes of the Go annotation analysis indicated that the DEGs were enriched in processes related to the immune system (Fig. 8B). Subsequently, KEGG pathway analysis revealed enrichment of these DEGs in pathways related to the immune system and infection (Fig. 8C). Finally, a GSEA was performed on all genes between the two immune patterns (Fig. 8D,E). The results revealed that eight BPs, such as the WNT signaling pathway and TGF BETA signaling pathway, were activated in Cluster 1 compared with Cluster 2. Simultaneously, eight BPs, such as antigen processing and presentation, NK cell‐mediated cytotoxicity, and primary immunodeficiency, were inhibited.

### Differences in immune characteristics

**Fig. 9:**
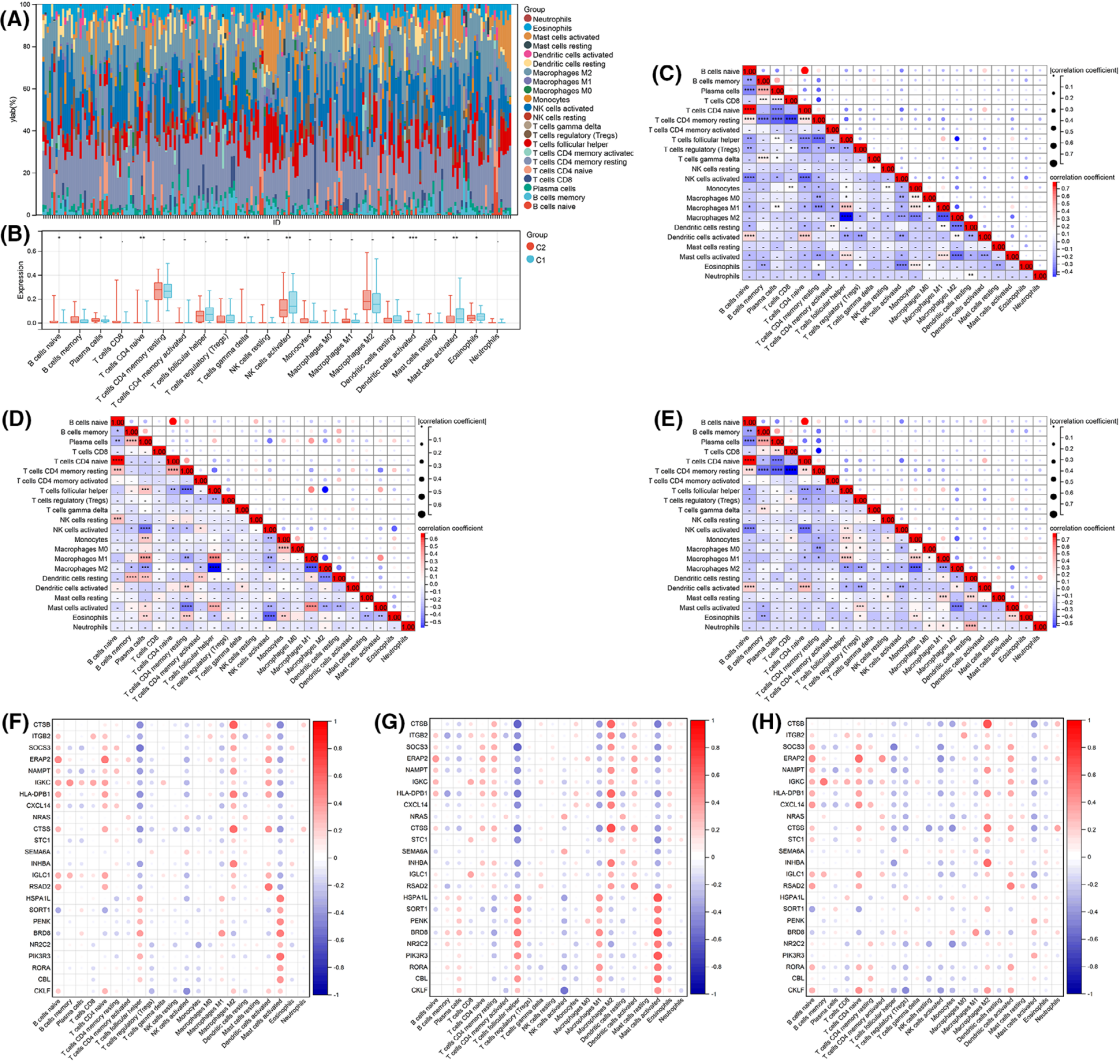
Immune characteristics between two different immune patterns. (A) Immune cell content stacking plot for each sample. (B) Twenty‐two immune cell types content histogram in two different immune patterns. Correlations between 22 immune cells in all patients (C), Clusters 1 patient (D), and Cluster 2 patients (E). Correlations between 24 immDEGs and immune cell types in all individuals (F), Cluster 1 patients (G), and Cluster 2 patients (H). Presentation of data as means ± standard deviation. *P < 0.05; **P < 0.01; ***P < 0.001; rank‐sum test.

Immune cell infiltration was estimated using CIBERSORTx. Figure 9A displays the proportion of 22 immune cell types in each sample. The result of unsupervised clustering of the samples with immune cell types is shown in Fig. S2B. Notably, the levels of activated NK cells, resting dendritic cells, activated mast cells, and eosinophils were remarkably elevated in Cluster 1 compared with Cluster 2. In contrast, the levels of naïve B cells, memory B cells, plasma cells, native CD4+ T cells, gamma delta T cells, and activated dendritic cells were considerably lowered in Cluster 1 than in Cluster 2 (Fig. 9B).

The correlation between the immune cell contents of samples in all individuals of Cluster 1 and Cluster 2 was calculated. In all individuals, a significant positive correlation was observed between naïve B cells and native CD4+ T cells, whereas the remaining cell types primarily showed negative correlations (Fig. 9C). In Cluster 1, which had a poor prognosis, naïve B cells exhibited a positive correlation with native CD4+ T cells. Moreover, M1 macrophages displayed a positive correlation with plasma cells, follicular helper T cells, and activated mast cells. In contrast, M2 macrophages showed a negative correlation with plasma cells, follicular helper T cells, M1 macrophages, and resting dendritic cells. Additionally, eosinophils exhibited a negative correlation with active NK cells (Fig. 9D). In Cluster 2, the correlation between immune cell contents was like that observed in all patients (Fig. 9E).

Finally, the correlations between 24 immDEGs and immune cell types were calculated in all patients (Fig. 9F), individuals in Cluster 1 (Fig. 9G), and individuals in Cluster 2 (Fig. 9H). Follicular helper T cells, macrophages, and activated mast cells had a remarkable association with immDEGs.

### Validation of the immDEGs risk signature in specific pathology types

**Fig. 10:**
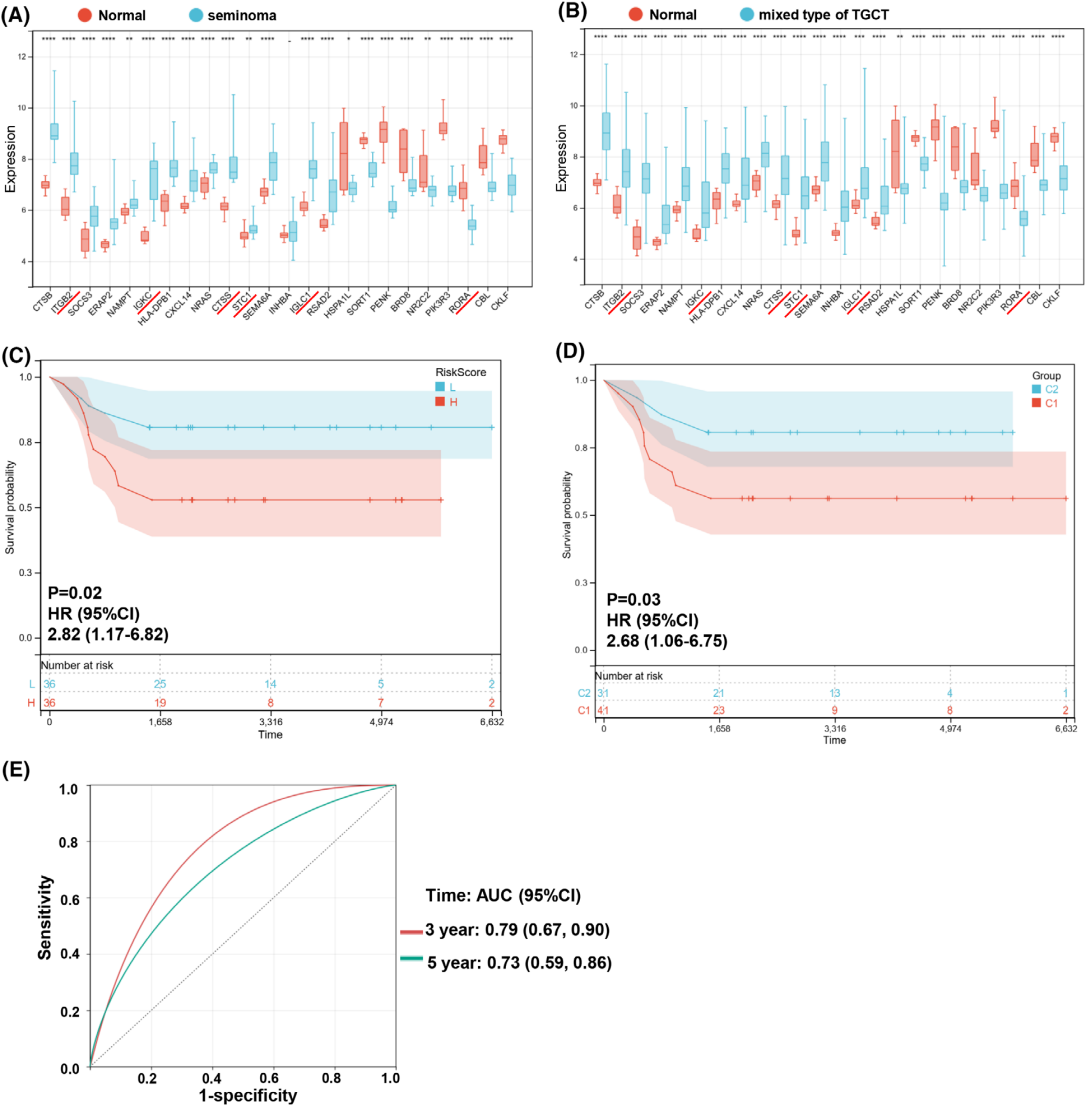
(A) Expression histogram of 24 immDEGs in seminoma (*n* = 26) and normal tissues (*n* = 12). (B) The expression histogram of 24 immDEGs in mixed type of TGCT (*n* = 90) and normal tissues (*n* = 12). (C) Kaplan–Meier survival analysis of the two risk groups, mixed type of TGCT samples were divided into high‐ and low‐risk groups based on their median risk score. (D) Kaplan–Meier survival analysis of the two immune molecular subtypes in mixed type of TGCT samples. (E) Time‐dependent ROC curves verified the predictive efficacy of the risk signature at 3 and 5 years in mixed type of TGCT samples. *P < 0.05; **P < 0.01; ****P < 0.0001; rank‐sum test.

TGCT is the most common type of testicular cancer. Seminoma is the most common histologic subtype of TGCT in young men. Mixed type of TGCT is the most common type of nonseminoma. Pure embryonal carcinoma, teratoma, and yolk sac tumor are rare. Therefore, we validated the validity of the six‐immDEGs risk signature in pure seminoma or mixed type of TGCT. The 24 immDEGs expression levels were significantly different between normal samples and seminoma or mixed type of TGCT (Fig. 10A,B). All seminoma samples belonged to the Cluster 2 subtype. Cluster 2 subtype possessed a favorable OS compared with Cluster 1. This is consistent with a better prognosis in patients with seminoma. Due to the lack of survival data in seminoma samples, we are unable to assess the prognostic value of the risk signature in patients with seminoma. For patients with mixed type of TGCT, KM survival curves revealed that the individuals in the high‐risk group possessed a poor OS (HR, 2.82; 95% CI, 1.17–6.82; *P* = 0.02; Fig. 10C). The 48 mixed type of TGCT samples belonged to Cluster 1, and 42 mixed type of TGCT samples belonged to Cluster 1. KM survival curves revealed that Cluster 1 possessed a poor OS (HR, 2.68; 95% CI, 1.06–6.75; *P* = 0.03; Fig. 10D). The AUC of Time‐dependent ROC at different time points were 0.79 (3 years) and 0.73 (5 years; Fig. 10E). This risk signature could serve as a predictor of survival for patients with mixed type of TGCT.

### Validation of STC1, RORA, and IGKC

**Fig. 11:**
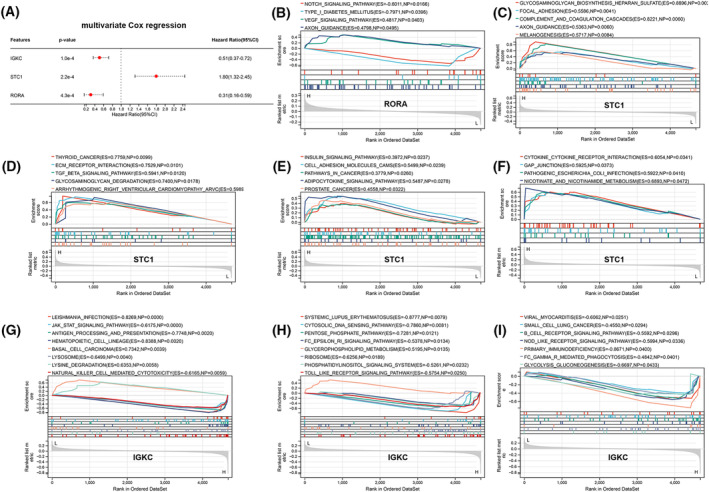
GSEA analysis for RORA, STC1, and IGKC. (A) Identification of three immDEGs related to OS of TGCT via multivariate Cox regression. (B) GSEA analysis for RORA. (C–F) GSEA analysis for STC1. (G–I) GSEA analysis for IGKC.

Through multivariate Cox regression analysis of the 24 immDEGs, IGKC, STC1, and RORA were identified as being considerably linked to the patient OS (Fig. 11A). GSEA was conducted between different gene expression groups that were significantly different in terms of BPs. The high‐*RORA* group showed inhibition of NOTCH signaling pathways and type‐1 diabetes mellitus, while the VEGF signaling pathway and axon guidance were activated in this group (Fig. 11B). Numerous tumor‐associated pathways were activated in the high‐*STC1* group (Fig. 11C–F). Numerous tumor‐associated pathways were also activated in the low‐*IGKC* group, and only basal cell carcinoma, lysine degradation, and glycerophospholipid metabolism were activated in the high‐*IGKC* group (Fig. 11G–I).

**Fig. 12:**
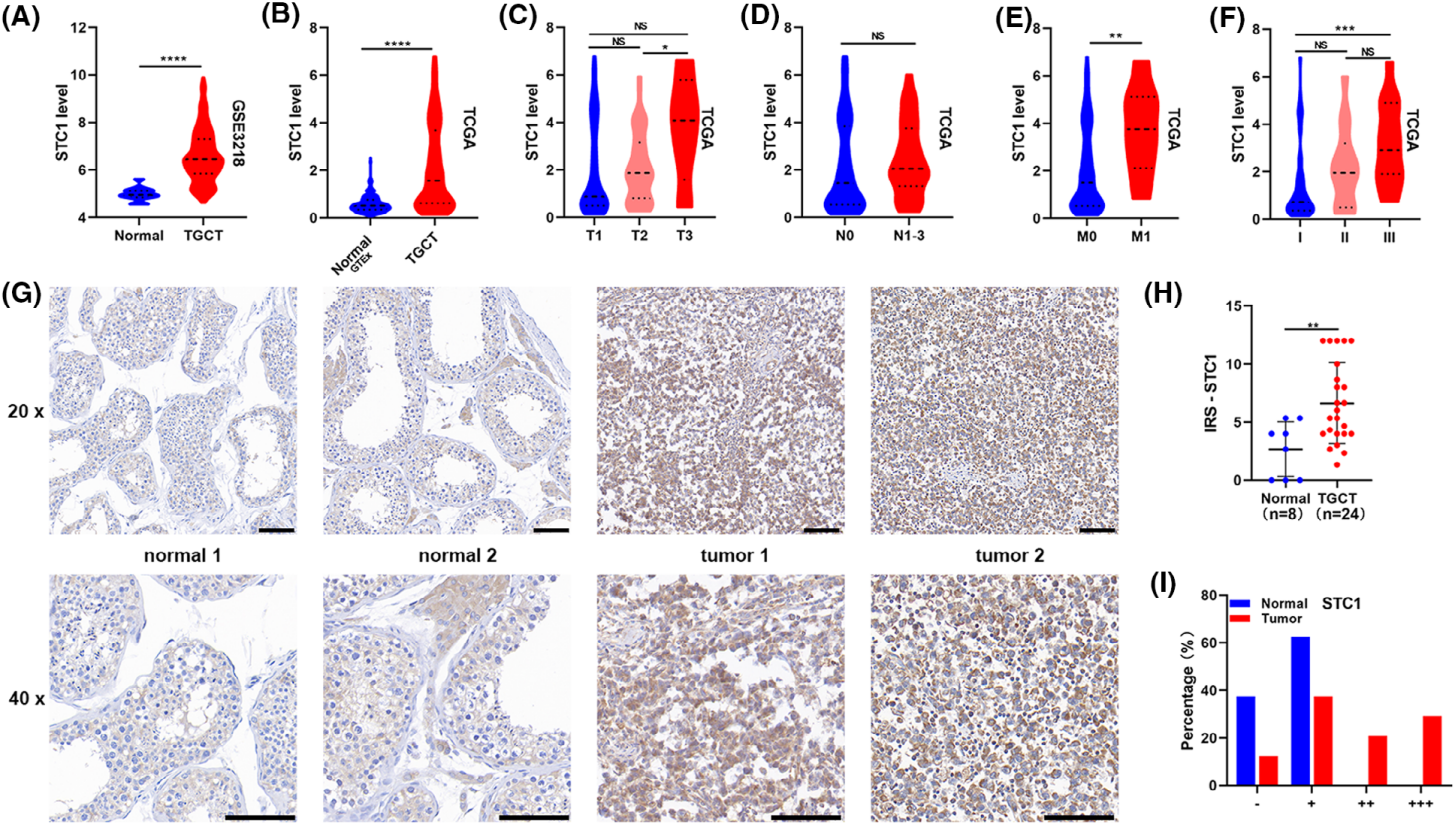
Elevated expression levels of STC1 positively correlated with advanced clinicopathological features and indicated poor prognosis. (A, B) The expression levels of STC1 increased in TGCT in GSE3218 and TCGA. (C–F) STC1 expression levels in TGCT tissues with different tumor stages, lymph node metastasis status, metastasis status, and grade status. (G) STC1 expressions in 32 clinical samples were detected by IHC. Scale bars: 100 μm. (H) Quantification of STC1 protein expression in TGCT tissues and normal tissues. (I) The proportion of clinical tissues with negative (−), weak (+), moderate (++), and strong (+++) STC1 staining intensity. Presentation of data as means ± standard deviation. ^NS^ *P* > 0.05; *P < 0.05; **P < 0.01; ***P < 0.001; ****P < 0.0001; rank‐sum test. T, tumor stage; N, lymph node metastasis; M, metastasis.

*STC1* was the only high‐risk factor for OS (HR, 1.80; 95% CI, 1.32–2.45; *P* < 0.001). STC1 in TGCT was further characterized through bioinformatics and IHC experiments. First, the expression of STC1 was evaluated in TGCT datasets (GSE3218, TCGA). As demonstrated in Fig. 12A,B, STC1 expression levels were elevated in TGCT tissues compared with its corresponding normal tissues. Moreover, STC1 was found to be upregulated in 32 out of 66 tumors in TCGA (Fig. S4). Subsequently, the correlation between STC1 expression and clinicopathological features was examined in TGCT patients from TCGA. STC1 expression was found to be correlated with clinical T, metastasis, and tumor stage. (Fig. 12C–F). Finally, the expression of STC1 was examined in clinical tissues by means of IHC. Results from IHC analysis of 24 seminoma tissues and eight normal tissues revealed a substantial increase in STC1 expression levels in TGCT samples (Fig. 12G–I).

## Discussion

Although testicular cancer is a curable tumor, it is also associated with primary resistance, disease progression after therapy, and adverse effects of treatment. Identifying underlying molecular mechanisms and markers of poor prognosis is important for this subset of patients. The TME has been regarded as a critical factor in influencing tumorigenesis and progression [24, 25]. In TME, immune cells constitute a significant proportion of nontumor cells. But a systematic understanding of the immune milieu in testicular cancer is currently lacking. Hence, exploring patterns of immune infiltration in testicular cancer and finding novel targets have important clinical benefits for early diagnosis and personalized therapy [26, 27, 28].

This study obtained 202 TCGT samples and 12 normal samples to identify immDEGs. A risk signature consisting of six immDEGs was constructed for TGCT for the first time. This signature was used to identify two immune molecular subtypes, namely Cluster 1 and Cluster 2. Prognostic differences were found to be significant between these two immune subtypes.

A total of 357 DEGs (tumor vs. normal) and 88 DEGs (Cluster 1 vs. Cluster 2) were screened, respectively. The 24 immDEGs were identified in TCGT samples compared with normal samples. In the PPI network of two sets of DEGs, the 24 immDEGs were not closely linked and scattered throughout the network. This suggested that their functions might be relatively independent. In GO and KEGG analyses, DEGs between tumor and normal were mainly enriched in the reproductive process. Studies have shown that TGCT can disrupt the hypothalamus‐pituitary‐gonadal axis and lead to obstruction of sperm production, which can be manifested as decreased sperm quality or azoospermia [29, 30, 31]. DEGs between the two immune patterns were enriched in various immune processes. Through GSEA, 16 BPs may affect the prognosis of TGCT. The pathways that have been extensively studied in different types of cancer, such as the WNT signaling pathway and TGF‐β signaling pathway, were also included. However, the studies of these two signaling pathways in testicular function and testicular cancer are limited. Young *et al*. [32] reported that the WNT pathway plays a role in normal spermatogenesis and that inhibiting canonical WNT signaling can attenuate the proliferation and migration ability of seminoma cells. *TCF7L1*, a WNT suppressor, can sensitively distinguish TGCT from nonseminomatous germ cell tumors [33]. The TGF‐β signaling pathway is essential for testis formation and can impact processes involved in testicular pathologies, including testicular cancer [34, 35]. Immune pathways mainly include antigen processing and presentation, natural killer (NK) cell‐mediated cytotoxicity, and primary immunodeficiency. Immunotherapy has revolutionized cancer treatment. The process of antigen presentation is the initial stage of the immune response and is critical for mounting an effective antitumor response [36]. It is well known that PDL1 expressed in tumor cell membranes binding to immune cell PD1 suppresses antitumor immunity [37]. The inhibitors of PD1/PDL1 have shown clinical efficacy in many tumors. TGCT has been the subject of various studies that indicate an elevation in PDL1 expression levels, and PD1/PDL1 is a potential therapeutic target of TGCT [9, 10, 11, 12, 13]. Cytotoxic T and NK cells are the main mediators of cytotoxicity against tumors [38]. By the directed release of lytic granules or by inducing apoptosis mediated by death receptors, NK cells can rapidly and directly kill tumor cells. Therefore, PD1/PDL1 inhibitors and NK cells have broad application prospects in TGCT, especially in patients who are resistant to conventional treatments or have relapsed.

A total of eight immDEGs (*IGKC*, *IGLC1*, *STC1*, *ITGB2*, *CTSS*, *RORA*, *HLA‐DPB1*, and *RSAD2*) were found to be related to OS of patients of TGCT via univariate Cox regression. However, the expressions of *IGKC*, *IGLC1*, *ITGB2*, *CTSS*, *HLA‐DPB1*, and *RSAD2* were elevated in tumor tissue and they served as protective factors. Such conflicting expression levels and prognostic value are not uncommon in bioinformatics analysis of tumors. I think this can be explained by the following reasons. First, unlike single‐cell sequencing, in this study, we obtained the average transcriptome data of a population of cells, not just tumor cells. The changes in gene expression may be due to nontumor cells. Or the gene exerts biological effects through nontumor cell pathways. For example, Cao *et al*. [39] reported that high expression of *CXCL11* in colon cancer could improve the prognosis of patients by promoting antitumor immunity. Second, we measured the mRNA expression levels of genes only at one point in time. The level of gene transcription is regulated by many factors and changes rapidly. Third, mRNA is subject to post‐transcriptional regulation. The mRNA expression levels are not equivalent to protein expression levels. Proteins are the main bodies that perform biological functions. The protein expression levels of genes need to be further verified.

Except for *HLA‐DPB1*, the remaining seven genes have not been studied in testicular cancer. According to Gotovac *et al*. [40], there was an observed increase in the frequency of the HLA‐DPB1*1701 allele in individuals diagnosed with TGCT, suggesting that the HLA region might have a function in the onset and progression of this tumor. Then, three genes (*IGKC*, *STC1*, and *RORA*) related to the OS of patients were identified via multivariate Cox regression by 24 immDEGs. In breast carcinoma, non‐small‐cell lung carcinoma, and colorectal carcinoma, IGKC has been linked to a favorable prognosis and may be utilized as a compatible prognostic indicator in human solid tumors [41, 42]. However, *IGKC* was significantly higher in ovarian cancer and clear cell renal cell carcinoma than in normal tissues [43, 44]. *RORA*, one of the circadian genes, inhibited tumorigenesis and progression in various tumors, including breast cancer [44], prostate cancer [45, 46], lung carcinoma [45, 47], endometrial cancer [48], and gastric cancer [49]. Only *STC1* behaves as an oncogene in our results. Stanniocalcin‐1 (*STC1*) promoted different types of cancer progression [50, 51, 52]. According to Lin *et al*. [53], STC1 is involved in immune evasion and resistance to immunotherapy and is negatively correlated with patient survival in various types of cancer. The expression level of STC1 in clinical tissue samples was examined and confirmed to be elevated in TGCT. GSEA revealed that numerous tumor‐associated pathways were activated in the high‐STC1 group. The mechanism by which STC1 promotes the initiation and progression of TGCT is still unknown and requires further study.

A novel immunophenotyping of TGCT was presented. Based on six signature immDEGs, two immune molecular subtypes (Cluster 1 and Cluster 2) were identified, and Cluster 1 possessed an unfavorable OS compared with Cluster 2. Traditional histological classification has certain limitations, especially for high‐grade tumors, chemotherapy‐resistant tumors, and recurrent tumors. In patients with the same histological classification, there may be large differences in the efficacy of the same treatment. The fast advancement of molecular technologies, such as sequencing, has led to an increasing application of molecular subtyping in various types of tumors [54, 55, 56]. Molecular subtyping is a promising way to provide precise personalized treatment and avoid unnecessary toxicities. There are 10 immune cell types that are differently expressed in the two molecular subtypes. Cluster 1 exhibited a significant reduction in T and B cells compared with Cluster 2. Combined with the pathway analysis, the worse prognosis of Cluster 1 might be the result of decreased ability of antigen presentation and the failure of activation of T cells and B cells leads to inhibiting antitumor immunity.

Seminoma is the most common histologic subtype of TGCT in young men. Mixed type of TGCT is the most common type of nonseminoma. Pure embryonal carcinoma, teratoma, and yolk sac tumor are rare. Medvedev *et al*. and Savelyeva *et al*. [57, 58] identified two distinct seminoma subtypes in 64 pure seminoma samples from TCGA. They found that seminoma subtype 2 shows signs of differentiation into nonseminoma TGCT and may have higher resistance to platinum‐based chemotherapy, higher immune score, and overexpression of 21 genes related to senescence‐associated secretory phenotype. We validated the validity of the six‐immDEGs risk signature in pure seminoma or mixed type of TGCT. All seminoma samples belonged to the Cluster 2 subtype. Cluster 2 subtype possessed a favorable OS compared with Cluster 1. This is consistent with a better prognosis in patients with seminoma. This six‐immDEGs risk signature could serve as a reliable predictor of survival for patients with mixed type of TGCT.

Although we got some meaningful results, there are some inherent limitations in our study. First, the study was based on single‐center data. This six‐immDEGs risk signature needs to be validated in more clinical cohorts. Second, we used a dataset that included seminoma, mixed type of TGCT, embryonal carcinoma, teratoma, and yolk sac tumor. Although we validated the six‐immDEG risk marker in pure seminoma and mixed type of TGCT, a different risk model might be obtained if one of the TGCT types were analyzed alone. Third, the genes identified in this study need to be experimentally verified to explore their mechanisms in TGCT.

To summarize, bioinformatics analyses were conducted to compare immune infiltration in the TGCT and normal samples. A six‐immDEGs risk signature was constructed, and two immune molecular subtypes with significant prognostic differences were identified. WNT signaling pathway, TGF‐β signaling pathway, antigen processing and presentation, and NK cell‐mediated cytotoxicity were linked to TGCT. The expression level of STC1 was found to be elevated in TCGA, GSE3218, and clinical tissues, and its high expression level was linked to advanced clinicopathological features and poor prognosis. These findings contribute to a better understanding of TGCT.

## Conflict of interest

The authors declare no conflict of interest.

## Author contributions

ZZ contributed to writing—original draft, funding acquisition, and resources. XX contributed to conceptualization and writing—review and editing. XW and MW contributed to methodology, software, and formal analysis. CM contributed to resources and writing—review and editing. ZL contributed to conceptualization, software, funding acquisition, and writing—review and editing.

## Supporting information


**Fig. S1.** Sample distribution before de‐batching (A) and after de‐batching (B).

**Fig. S2.** Unsupervised clustering of the samples with all genes (A) or 22 immune cell types (B).

**Fig. S3.** Screening out six characteristic genes out of eight immDEGs using the LASSO algorithm. (A) LASSO coefficient path diagram. (B) LASSO regression analysis cross validation curve.

**Fig. S4.** Pan‐cancer analysis of STC1. ‐ P > 0.05; *P < 0.05; **P < 0.01; ***P < 0.001; rank‐sum test. Tumor types (sample number): ACC (tumor = 79, normal = 0, GTEx = 258); BLCA (tumor = 406, normal =19, GTEx = 21); BRCA (tumor = 1101, normal = 113, GTEx = 459); CEST (tumor = 306, normal = 3, GTEx = 19); CHOL (tumor = 35, normal = 9); COAD (tumor = 455, normal = 41, GTEx = 779); DLBC (tumor = 48, normal = 0, GTEx = 929); ESCA (tumor = 163, normal = 11, GTEx = 1445); GBM (tumor = 153, normal = 5, GTEx = 2642); HNSC (tumor = 504, normal = 44); KICH (tumor = 65, normal = 25, GTEx = 89); KIRC (tumor = 532, normal = 72, GTEx = 89); KIRP (tumor = 290, normal = 32, GTEx = 89); LAML (tumor = 150, normal = 0); LGG (tumor = 513, normal = 0, GTEx = 2642); LIHC (tumor = 371, normal = 50, GTEx = 226); LUAD (tumor = 516, normal = 59, GTEx = 578); LUSC (tumor = 501, normal = 49, GTEx = 623); MESO (tumor = 87, normal = 0); OV (tumor = 376, normal = 0, GTEx = 180); PAAD (tumor = 179, normal = 4, GTEx = 328); PCPG (tumor = 181, normal = 3); PRAD (tumor = 498, normal = 52, GTEx = 245); READ (tumor = 165, normal = 10, GTEx = 779); SARC (tumor = 260, normal = 2); SKCM (tumor = 471, normal = 1, GTEx = 1809); STAD (tumor = 375, normal = 32, GTEx = 359); TGCT (tumor = 134, normal = 0, GTEx = 391); THCA (tumor = 512, normal = 59, GTEx = 653); THYM (tumor = 120, normal = 2); UCEC (tumor = 545, normal = 35, GTEx = 142); UCS (tumor = 57, normal = 0, GTEx = 142); UVM (tumor = 80, normal = 0).

## Data Availability

The data that support the findings of this study are openly available in GEO and TCGA databases.
