# Supplementary figures and images for: Identification of testicular cancer immune infiltrates and novel immune cell subtypes
Source: FEBS Open Bio. 2023 Aug 10;13(10):1967–85. doi: 10.1002/2211-5463.13688 (PMC10549230; doi:10.1002/2211-5463.13688)

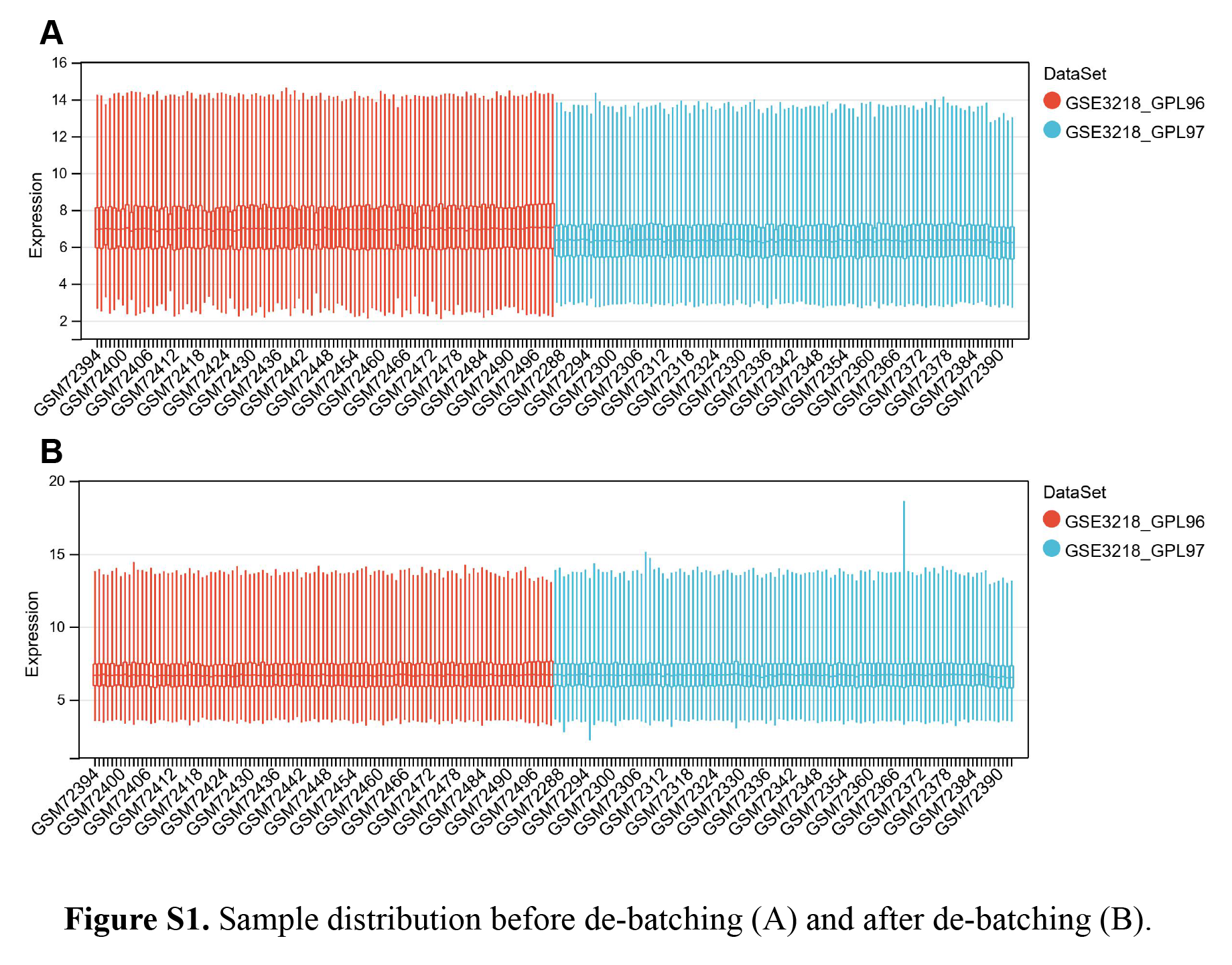

Supplement: Supplementary file 1 — Fig. S1. Sample distribution before de‐batching (A) and after de‐batching (B). [file FEB4-13-1967-s003.tif]

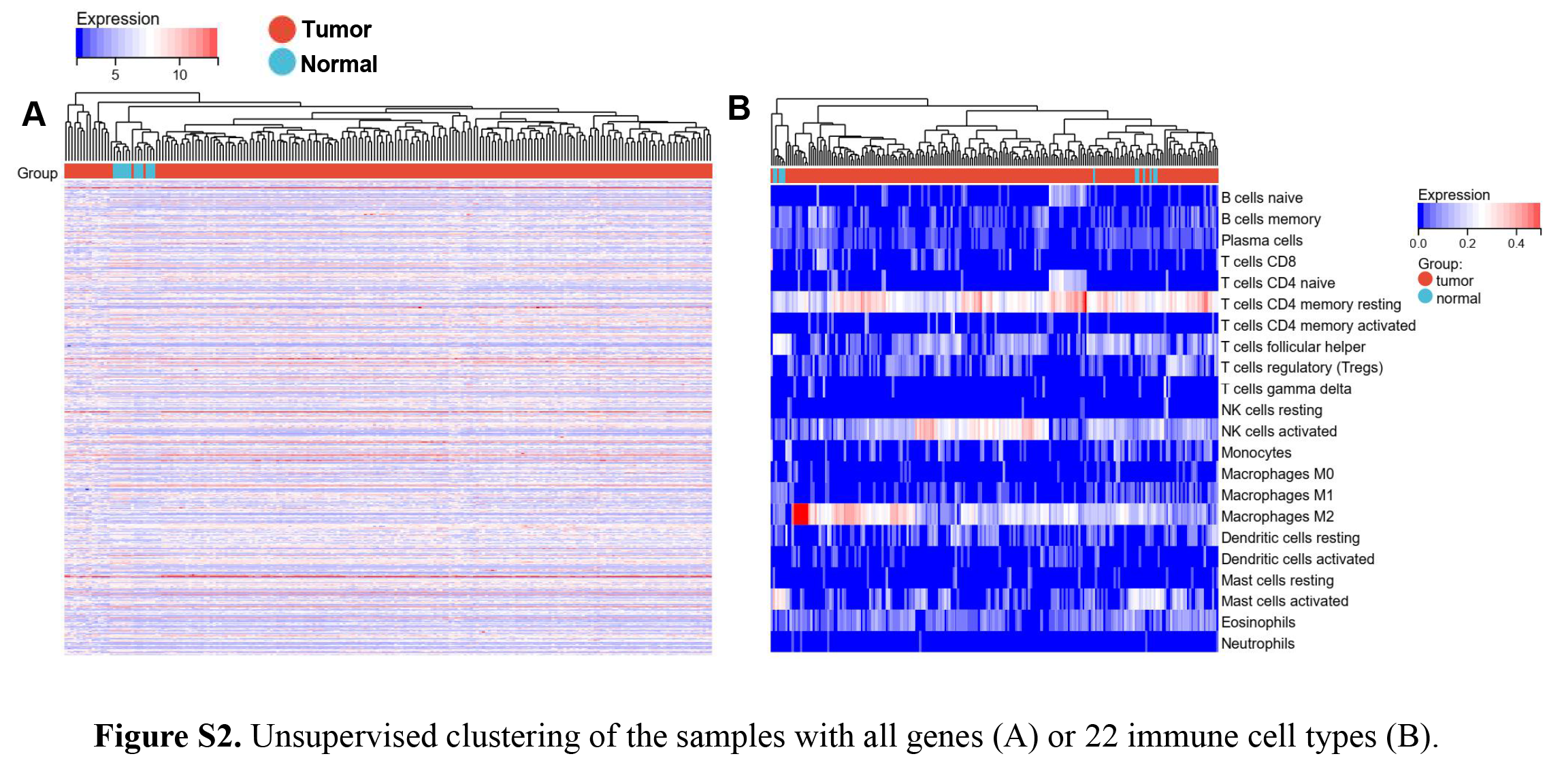

Supplement: Supplementary file 2 — Fig. S2. Unsupervised clustering of the samples with all genes (A) or 22 immune cell types (B). [file FEB4-13-1967-s001.tif]

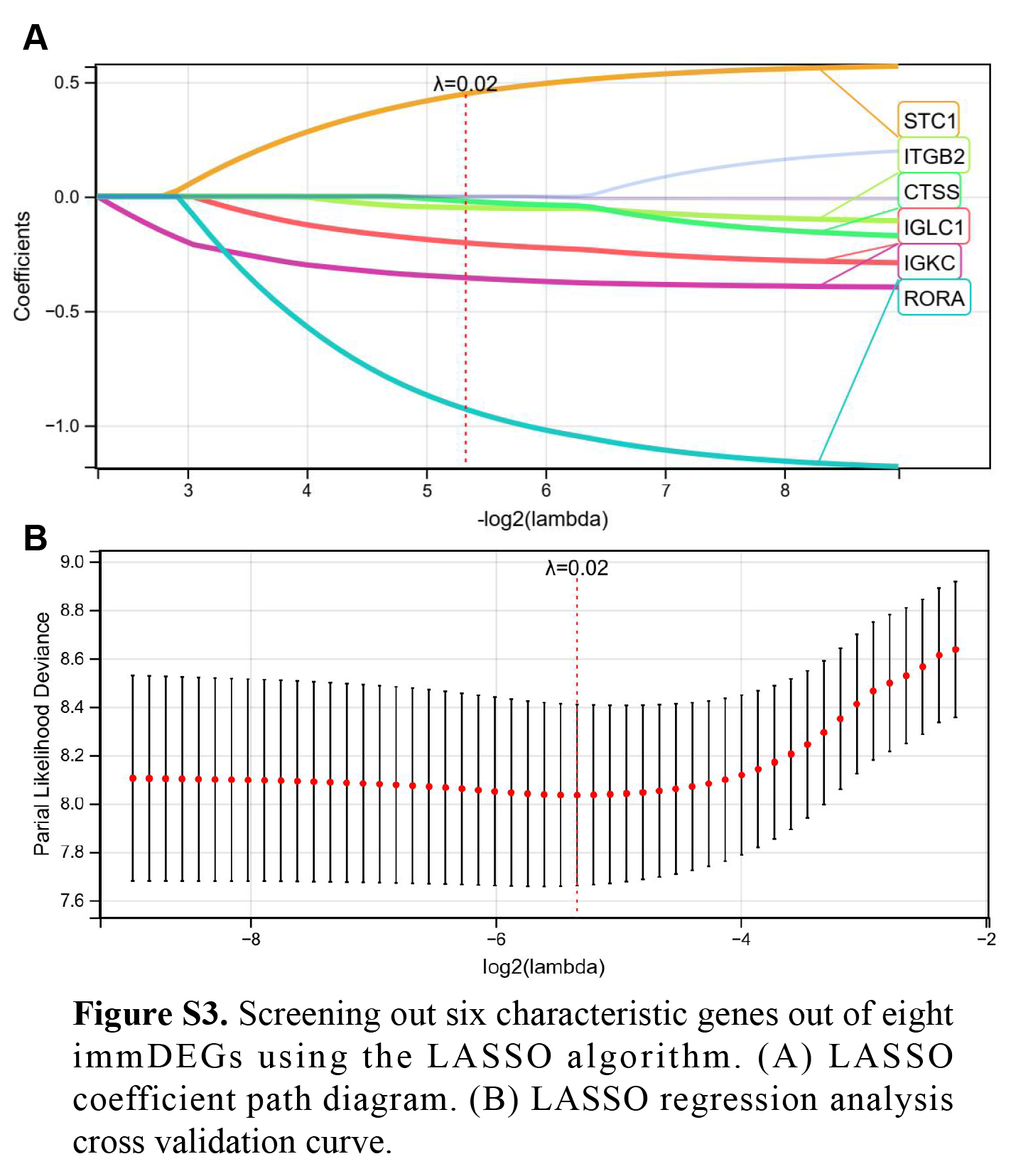

Supplement: Supplementary file 3 — Fig. S3. Screening out six characteristic genes out of eight immDEGs using the LASSO algorithm. (A) LASSO coefficient path diagram. (B) LASSO regression analysis cross validation curve. [file FEB4-13-1967-s004.tif]
