# Supplementary material for: Identification of testicular cancer immune infiltrates and novel immune cell subtypes
Source: FEBS Open Bio. 2023 Aug 10;13(10):1967–85. doi: 10.1002/2211-5463.13688 (PMC10549230; doi:10.1002/2211-5463.13688)
Supplement: Supplementary file 4 — Fig. S4. Pan‐cancer analysis of STC1. ‐ P > 0.05; *P < 0.05; **P < 0.01; ***P < 0.001; rank‐sum test. Tumor types (sample number): ACC (tumor = 79, normal = 0, GTEx = 258); BLCA (tumor = 406, normal =19, GTEx = 21); BRCA (tumor = 1101, normal = 113, GTEx = 459); CEST (tumor = 306, normal = 3, GTEx = 19); CHOL (tumor = 35, normal = 9); COAD (tumor = 455, normal = 41, GTEx = 779); DLBC (tumor = 48, normal = 0, GTEx = 929); ESCA (tumor = 163, normal = 11, GTEx = 1445); GBM (tumor = 153, normal = 5, GTEx = 2642); HNSC (tumor = 504, normal = 44); KICH (tumor = 65, normal = 25, GTEx = 89); KIRC (tumor = 532, normal = 72, GTEx = 89); KIRP (tumor = 290, normal = 32, GTEx = 89); LAML (tumor = 150, normal = 0); LGG (tumor = 513, normal = 0, GTEx = 2642); LIHC (tumor = 371, normal = 50, GTEx = 226); LUAD (tumor = 516, normal = 59, GTEx = 578); LUSC (tumor = 501, normal = 49, GTEx = 623); MESO (tumor = 87, normal = 0); OV (tumor = 376, normal = 0, GTEx = 180); PAAD (tumor = 179, normal = 4, GTEx = 328); PCPG (tumor = 181, normal = 3); PRAD (tumor = 498, normal = 52, GTEx = 245); READ (tumor = 165, normal = 10, GTEx = 779); SARC (tumor = 260, normal = 2); SKCM (tumor = 471, normal = 1, GTEx = 1809); STAD (tumor = 375, normal = 32, GTEx = 359); TGCT (tumor = 134, normal = 0, GTEx = 391); THCA (tumor = 512, normal = 59, GTEx = 653); THYM (tumor = 120, normal = 2); UCEC (tumor = 545, normal = 35, GTEx = 142); UCS (tumor = 57, normal = 0, GTEx = 142); UVM (tumor = 80, normal = 0). [file FEB4-13-1967-s002.pdf]

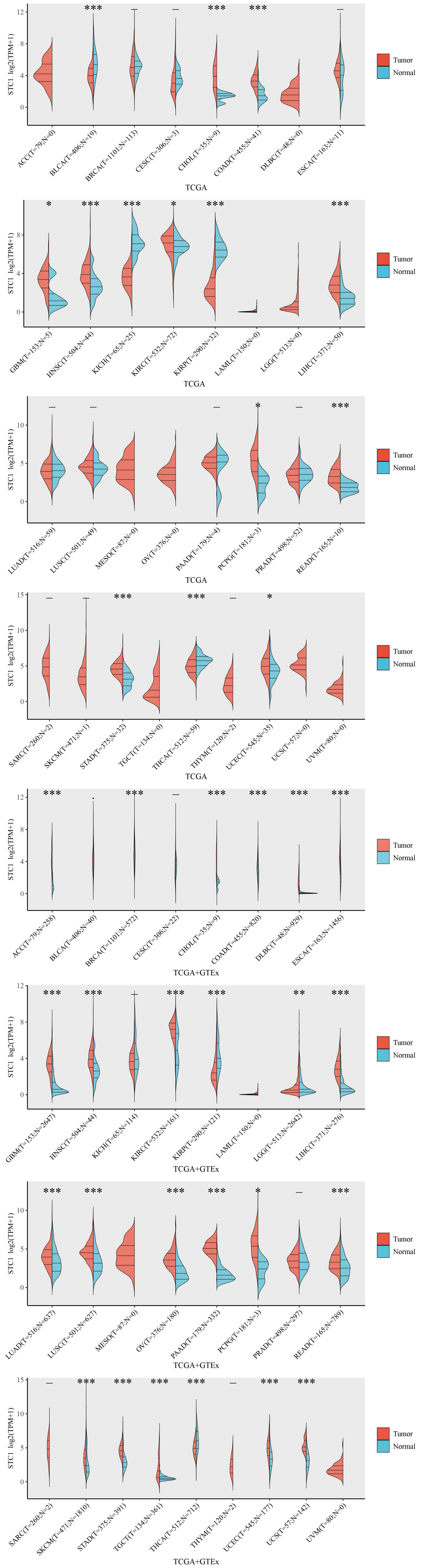

Figure S4. Pan-cancer analysis of STC1. - P> 0.05; *P < 0.05; **P < 0.01; ***P < 0.001; rank-sum test. Tumor types (sample number): ACC (tumor=79, normal=0, GTEx=258); BLCA (tumor=406, normal=19 , GTEx=21); BRCA (tumor=1101, normal=113 , GTEx=459); CEST (tumor=306, normal=3 , GTEx=19); CHOL (tumor=35, normal=9); COAD (tumor=455, normal=41 , GTEx=779); DLBC (tumor=48, normal=0 , GTEx=929); ESCA (tumor=163, normal=11 , GTEx=1445); GBM (tumor=153, normal=5 , GTEx=2642); HNSC (tumor=504, normal=44); KICH (tumor=65, normal=25 , GTEx=89); KIRC (tumor=532, normal=72 , GTEx=89); KIRP (tumor=290, normal=32 , GTEx=89); LAML (tumor=150, normal=0); LGG (tumor=513, normal=0 , GTEx=2642); LIHC (tumor=371, normal=50 , GTEx=226); LUAD (tumor=516, normal=59 , GTEx=578); LUSC (tumor=501, normal=49 , GTEx=623); MESO (tumor=87, normal=0); OV (tumor=376, normal=0 , GTEx=180); PAAD (tumor=179, normal=4 , GTEx=328); PCPG (tumor=181, normal=3); PRAD (tumor=498, normal=52 , GTEx=245); READ (tumor=165, normal=10 , GTEx=779); SARC (tumor=260, normal=2); SKCM (tumor=471, normal=1 , GTEx=1809); STAD (tumor=375, normal=32 , GTEx=359); TGCT (tumor=134, normal=0 , GTEx=391); THCA (tumor=512, normal=59 , GTEx=653); THYM (tumor=120, normal=2); UCEC (tumor=545, normal=35 , GTEx=142); UCS (tumor=57, normal=0 , GTEx=142); UVM (tumor=80, normal=0).
